# High-intensity care for GDMT titration

**DOI:** 10.1007/s10741-024-10419-5

**Published:** 2024-07-22

**Authors:** Jan Biegus, Matteo Pagnesi, Beth Davison, Piotr Ponikowski, Alexander Mebazaa, Gadi Cotter

**Affiliations:** 1 Institute of Heart Diseases, Wroclaw Medical University, 50-556 Wroclaw, Borowska 213 Poland; 2 Cardiology, ASST Spedali Civili and Department of Medical and Surgical Specialties, Radiological Sciences, and Public Health, University of Brescia, Brescia, Italy; 3 Momentum Research Inc, Durham, NC USA; 4 Heart Initiative, Durham, NC USA; 5 Université Paris Cité, INSERM UMR-S 942(MASCOT), Paris, France; 6 Department of Anesthesiology and Critical Care and Burn Unit, Saint-Louis and Lariboisière Hospitals, FHU PROMICE, DMU Parabol, APHP Nord, Paris, France

**Keywords:** GDMT (guideline-directed medical therapy), Pharmacotherapy, Up-titration, GDMT optimization

## Abstract

Heart failure (HF) is a systemic disease associated with a high risk of morbidity, mortality, increased risk of hospitalizations, and low quality of life. Therefore, effective, systemic treatment strategies are necessary to mitigate these risks. In this manuscript, we emphasize the concept of high-intensity care to optimize guideline-directed medical therapy (GDMT) in HF patients. The document highlights the importance of achieving optimal recommended doses of GDMT medications, including beta-blockers, renin–angiotensin–aldosterone inhibitors, mineralocorticoid receptor antagonists, and sodium-glucose cotransporter inhibitors to improve patient outcomes, achieve effective, sustainable decongestion, and improve patient quality of life. The document also discusses potential obstacles to GDMT optimization, such as clinical inertia, physiological limitations, comorbidities, non-adherence, and frailty. Lastly, it also attempts to provide possible future scenarios of high-intensive care that could improve patient outcomes.

## Introduction

Heart failure (HF) is a systemic disease associated with a high risk of morbidity and mortality, thus needing effective, systemic treatment strategies [1, 2]. Although the pathophysiology of HF is complex and multi-factorial, it has been suggested that neurohormonal, adrenergic, and inflammatory activation may play a critical role in the disease, driving both its main symptoms and signs (congestion) as well as its adverse outcomes [3, 4]. Several effective pharmacological interventions have been discovered in the last decades, mostly modulating neurohormonal and adrenergic activation, which have dramatically improved the prognosis of patients with HF [5]. There are several drugs that make up guideline-directed medical therapy (GDMT), including beta-blockers (BBs), RASi (renin–angiotensin–aldosterone inhibitors) including ACEi (Angiotensin-Converting Enzyme Inhibitor), ARB (Angiotensin Receptor Blocker) or ARNi (Angiotensin Receptor-Nephrilysin inhibitor), MRA (Mineralocorticoid Receptor Antagonist), and SGLT (sodium-glucose cotransporter) inhibitors, that have an undisputedly proven positive clinical effect, especially among patients with HF with reduced ejection fraction (HFrEF) [1, 2]. Importantly, when administering these medications, one has to consider that full effectiveness is reached when obtaining the optimal recommended drug doses [1, 2]. Although the dose–response effect of GDMT medications is not well examined, there is growing evidence that patients benefit from the treatment the most once the maximally tolerated dose of the drug is achieved. This article will discuss the benefits and challenges of strategies that aim to rapidly optimize the pharmacological treatment of HF patients, which is a “high-intensity care” approach.

## Obstacles in fast and effective initiation and up-titration of GDMT

There are several reasons why optimizing HF pharmacotherapy could be challenging from a clinical perspective. HF patients are often considered vulnerable and fragile, with multiorgan dysfunction and a high risk of adverse events related to the disease and its treatment [6–8]. A combination of clinical inertia and physiological limitations related to clinical parameters (blood pressure, heart rate, renal function, electrolytes), comorbidities, cost and socioeconomic status, non-adherence, and frailty are common barriers to full GDMT implementation [9–14]. For many years, physicians followed the unwritten rule that suggested slow and cautious up-titration of GDMT components to minimize the risk of any potential complications. Some of these barriers (cost, socioeconomic status) are difficult to modify, but others can be improved. Most importantly, the perception is that the HF treatment itself is related to a high risk of adverse events, especially when compared to treatment of other deadly diseases like cancers [15, 16]. In this respect, there is growing evidence that GDMT is safe and beneficial in the most vulnerable HF populations with perfectly acceptable risk profiles [17–21]. One can only imagine the difference between chemotherapy’s most difficult adverse effects, such as bone marrow suppression, lethal infections, nausea, hair loss, etc., and the less than 5% of the risk of developing mild hypotension, asymptomatic increases in potassium and creatinine, and bradycardia leading mostly to mild dizziness associated with high-intensity GDMT in HF. Moreover, the sequential discoveries of subsequent components of current GDMT and the way those components were tested in landmark clinical trials (on top of the standard of care at the time of their discovery) have introduced another bias related to the need for a sequential initiation of the drugs. However, it is estimated that if that approach is followed, it may take up to 2–3 months to reach the goal doses of all GDMT components, during which time the patient is undertreated and exposed to the risk of a possibly fatal adverse event, especially after an acute heart failure admission. The lack of prospective clinical trials comparing different strategies for optimizing HF pharmacotherapy has been another reason for such staged approaches over the years. Of note, the lack or scarcity of direct data showing that proactive up-titration of GDMT components, as well as other aspects of optimization of HF care, which may be called high-intensity care, may be beneficial in HF patients (or even critically needed), has also created a situation of great pharmacological inertia, as physicians did not have a stimulus to actively up-titrate GDMT, especially in the so-called stable patient [22]. Therefore, the prescription of high doses of GDMT to patients with HF by their treating physician is the first step to achieving such treatment.

Beyond drug prescription, many other barriers exist to patients being treated by high-intensity GDMT. Medication non-adherence is a big, unrecognized challenge. This problem has not been studied in detail; however, recent smaller studies and sub-analysis of large ones have suggested that nonadherence is common, associated with adverse outcomes, and potentially treatable by a multi-disciplinary strategy. Thus, tailored interventions need to be implemented to improve medication adherence [23].

Finally, cost and socioeconomic factors also limit high-intensity implementation in some parts of the developed world and may underdeveloped regions; those should also be addressed when implementing high-intensity care [13, 24].

## High-intensity care for HF patients

As mentioned above, high-intensity care can be defined as the proactive optimization of HF care. There are various ways to address the HF burden, including both pharmacological and non-pharmacological interventions. Several studies have demonstrated that different interventions that prioritize the care of HF patients can lead to better outcomes, such as improved survival rates and lower risk of hospitalization. Some of these interventions include optimizing pharmacotherapy and device therapy, administering vaccinations, providing nurse care, addressing medication cost issues and non-adherence, and utilizing remote monitoring [25–27]. Therefore, it is necessary to create mechanisms of patient care (high-intensity care) and patient care systems that will ensure optimal treatment and appropriate safety of the patient.

## What is the optimal dose of GDMT?

Most HF specialists now recognize the benefits of rapid and simultaneous up-titration of foundation therapy for HF, moving away from a sequential approach to GDMT optimization. In terms of pharmacotherapy, the goal of high-intensity care is to achieve the target (guideline-recommended) doses of four pillars of life-saving therapy while maintaining appropriate safety. ESC (European Society of Cardiology), Heart Failure Society of America (HFSA), and ACC (American College of Cardiology)/AHA (American Heart Association)/ Heart Failure Society of America (HFSA) guidelines provide the target doses of the main drug groups that are recommended for HFrEF (Table 1) [1, 2], with which most national guidelines agree. The safe optimization of GDMT can occur in ambulatory settings and during hospitalization. Thus, one needs to keep in mind that a hospital stay may be an optimal opportunity for initiating and optimizing the GDMT, as this time has several advantages, but if that opportunity is missed, it can have deleterious consequences [28, 29].

**Table 1:** Optimal recommended doses of GDMT in recent ESC and ACC/AHA/HFSA guidelines

**Medication generic name**	**ESC 2021 Target Dose**	**ACC/AHA/HFSA 2022 Target Dose**
**MRA**		
Eplerenone	50 mg o.d.	50 mg once daily
Spironolactone	50 mg o.d.	25–50 mg once daily
**Beta-blocker**		
Bisoprolol	10 mg o.d.	10 mg once daily
Carvedilol	25 mg b.i.d., 50 mg t.i.d. if patient >85 kg	25–50 mg twice daily
Carvedilol CR	--	80 mg once daily
Metoprolol succinate extended-release tablet	200 mg o.d.	200 mg once daily
Nebivolol	10 mg o.d.	--
**ACEi**		
Captopril	50 mg t.i.d.	50 mg 3 times daily
Enalapril	10_20 mg b.i.d.	10–20 mg twice daily
Lisinopril	20_35 mg o.d.	20–40 mg once daily
Ramipril	5 mg b.i.d.	10 mg once daily
Trandolapril	4 mg o.d.	4 mg once daily
Perindopril	--	8–16 mg once daily
Fosinopril	--	40 mg once daily
Quinopril	--	20 mg twice daily
**ARB**		
Candesartan	32 mg o.d.	32 mg once daily
Valsartan	160 mg b.i.d.	160 mg twice daily
Losartan	150 mg o.d.	50–150 mg once daily
**ARNi**		
Sacubitril/valsartan (Entresto^™^)	97/103 mg b.i.d.	97 mg sacubitril and 103 mg valsartan twice daily
**SGLT-2 Inhibitor**		
Dapagliflozin	10 mg o.d.	10 mg once daily
Empagliflozin	10 mg o.d.	10 mg once daily

## Mean doses of GDMTs in the landmark HF trials

Importantly, in many clinical trials that examined current GDMT, the target dose of the drugs in question was achieved in more than half of the study populations. In the SOLVD-Treatment trial, the mean dose of enalapril achieved in the study arm was 8.4 mg twice daily, with 49% of participants on 10 mg twice daily [30]. In the MERIT-HF trial, the mean dose of metoprolol was 159 mg per day, with 64% of patients on the target dose [31]. Analogously, in the CIBIS II trial, 63% of patients were on 10 mg of bisoprolol daily, with a mean achieved dose of 8.5 mg/day [32]. Similar results were achieved in COPERNICUS (65% of patients on target dose of Carvedilol) and SENIORS (68% of patients on target dose of nebivolol) [33, 34]. In the EPHESUS (Eplerenone Post–Acute Myocardial Infarction Heart Failure Efficacy and Survival Study) trial, the mean dose of eplerenone was 43.5 mg [35]. In the EMPHASIS-HF (Eplerenone in Mild Patients Hospitalization and Survival Study in Heart Failure) trial, 60.2% of patients at 5 months were on 50 mg of eplerenone, and the mean dose of the drug was 39 mg/day [36]. In PARADIGM-HF (Prospective Comparison of ARN Inhibitors With ACE Inhibitors to Determine Impact on Global Mortality and Morbidity in HF Trial), the mean dose of sacubitril/valsartan at the last study assessment was 375 ± 71 mg daily (the target dose was 400 mg per day) [37]. Since SGLT-2 inhibitors have only one recommended dose, all patients who tolerate the drug are on the target dose.

## Real-world GDMT utilization

The CHAMP-HF (Change the Management of Patients with Heart Failure) registry in the US collected data from over 150 cardiology practices across the country and included more than 3500 ambulatory HFrEF patients who were taking at least one oral HF medication at the time of enrollment [38]. Shockingly, approximately 1 out of 3 eligible patients were not on any RASi or beta-blocker, and 2 out of 3 were not receiving MRA. There was significant under-dosing of GDMT as the recommended doses were achieved in the following percentage of patients: ACEi/ARB 17%, ARNI 14%, beta-blockers 28%, and MRA 77%. Only 1.1% of eligible patients were simultaneously receiving target doses of all three GDMT classes [38].

The PINNACLE (Practice Innovation and Clinical Excellence) registry, which included over 6 million patients, reported trends in GDMT utilization among 700,000 HFrEF patients from 2013 to 2017 [39]. The positive aspect of the report is that the percentages of patients on beta-blockers and RASi were increasing over the analyzed years. Despite that, 75% were at least receiving a beta-blocker, 78% were at least receiving an ACEi/ARB/ARNI, and only 73% were receiving both a beta-blocker and any RASi [39].

Similarly, in a recent report from the ESC-HFA EORP (EURObservational Research Programme of the ESC) Heart Failure Long-Term Registry, the percentage of patients that were receiving any dose of beta-blocker, RASi, and MRA paradoxically decreased by approximately 12–14% each during the first year after the hospital discharge. Importantly, at one year, only 30% of patients received target doses of ACEi/ARB, and only 20% were on the recommended dose of beta-blockers [40]. In the Multinational ASIAN-HF registry, which enrolled HF patients between 2012 and 2015, the recommended doses of GDMT were achieved in only 17% for ACEi/ARB, 13% for beta-blockers, and 29% for MRA [41].

In a multinational (US, UK, and Sweden) observational study of patients who had initiated GDMT after hospitalization for HF, the target dose within the year was very low. Among the new users of ACEi (n = 8426), ARB (n = 2303), beta-blockers (n = 10,476), and ARNI (n = 29,546) over 12 months of follow-up, the target dose was achieved in only 15%, 10%, 12%, and 30%, respectively. Out of patients who started MRA (n = 17,421), only 5% had the dose up-titrated during the following year [42]. Of note, the discontinuation rates of GDMT were surprisingly high in that cohort, reaching 50% for ACEi [42].

The recently announced results of TITRATE-HF, a real-world multicentre longitudinal registry conducted in the Netherlands, have demonstrated that only 44% of patients are on quadruple therapy, with only 1% being on target doses of all GDMT [43, 44].

## The role of GDMT in HF

Each individual component of the current HF treatment has proven its efficacy in prospective RCTs in addition to previously proven therapies at the time. Each GDMT has a life-saving, additive effect that becomes apparent within weeks of initiation. Therefore, it is crucial to aim for maximally tolerated or target doses of all drug classes as quickly as possible [45]. The potential gain of the GDMT in HF is enormous, especially when compared to other deadly diseases. Based on clinical trials in HFrEF, it has been estimated that 55-year-old patients who received all four drug classes of GDMT had the potential to gain an additional 6.3 (3.4–9.1) years of life compared to those who only received an ACEi/ARB and beta-blocker [45]. The older patients had lower (but still significant) projected benefits, approximately 4.4 (2.5–6.2) years for 65-year-old patients [45]. This highlights the significance of high-intensity care in improving patient outcomes and emphasizes the importance of following the medical guidelines for optimal treatment.

The data regarding the efficacy of individual groups of medications in HF are most compelling for patients HFrEF patients. A meta-analysis of data from the most important HF trials (n = 75, with 95,444 participants) has revealed that the combination of ARNi, beta-blockers, MRA, and SGLT2i was most effective in reducing all-cause death (HR: 0.39; 95% CI: 0.31–0.49), when compared to patients on placebo [46]. The additional number of life-years gained for a 70-year-old patient on the most effective GDMT combination was estimated to be 5.0 (2.5–7.5) years compared with no treatment [46]. The evidence of the efficacy of single components of GDMT in patients with EF > 40% is much weaker. However, when the aggregate benefit of GDMT was estimated (from 13 studies, with 29,875 participants) in patients with HFmrEF and HFpEF, it turned out that a combination of ARNI, beta-blockers, MRA, and SGLT2i was the most effective (HR: 0.47 95% CI: 0.31–0.70); this was largely explained by the triple combination of ARNI, MRA, and SGLT2i [47]. The benefit was higher in patients with lower ejection fraction [47]. A recent analysis of newly diagnosed HFrEF patients hospitalized in the Get With The Guidelines–Heart Failure registry between 2016 and 2023 demonstrated that 83% out of 33,036 patients were eligible for quadruple therapy, while 93% were eligible for three components of GDMT. Importantly, however, between the years 2012–2023, when GDMT was already comprised of four components, only 15.3% were prescribed quadruple therapy, and 41.5% were prescribed triple therapy [48]. The projected benefit of implementation of GDMT was reaching 24.8% in absolute risk reduction when compared to no GDMT treatment [48].

## Rapid simultaneous vs step-by-step approach in initiation and optimization of pharmacotherapy in HF

As previously mentioned, the traditional approach to initiating and optimizing GDMT for HF involved a sequential initiation of medications based on the order of introduction of each individual molecule. This meant that treatment was often started with ACEi or ARBs (with gradual up-titration), followed by the addition of a beta-blocker and MRA if the patient was still experiencing symptoms. However, in 2016, the ESC guidelines were updated to recommend starting and up-titrating ACEi and beta-blockers simultaneously, with the addition of an MRA being recommended for symptomatic patients [49]. The approach has evolved to the rapid initiation and up-titration of four pillars of HF treatment almost simultaneously. When the traditional sequence of pharmacotherapy initiation has been compared to a more rapid approach, it has been demonstrated that the accelerated approach overperformed the conservative approach, with 23 fewer patients per 1000 patients experiencing a composite of HF hospitalization or cardiovascular death and 7 fewer deaths [50].

## The benefit of high-intensity care after hospitalization for acute HF

The STRONG-HF (Safety, tolerability, and efficacy of up-titration of guideline-directed medical therapies for acute heart failure) study was a prospective, randomized study that compared high-intensity care (HIC) vs. usual care (UC) after hospitalization for acute HF (AHF). The aim of the study was to assess the safety and efficacy of early optimization of oral HF therapy with beta-blockers, RASi, and MRA (HIC) along with close follow-up on the risk of the composite of all-cause mortality or HF readmission at 180 days [51]. After the initial stabilization, AHF patients who were not on optimal doses of neurohormonal therapies were randomized to either HIC, with GDMT initiation before discharge and the goal of achieving optimal GDMT doses by two weeks after discharge vs. UC. The data safety and monitoring board terminated the study prematurely due to the evident benefit of HIC. The primary composite endpoint occurred in 15.2% of patients in the HIC group when compared to 23.3% of UC, with a risk ratio of 0.66 (95% confidence interval 0.50–0.86). The targeted doses of GDMT were achieved by 90 days in a significantly higher percentage of patients in the HIC group vs. UC group: 49% vs. 4% for beta-blockers, 55% vs. 2% for RASi, and 84% vs. 46% for MRA, respectively [52]. Patients with low blood pressure, higher NYHA class as well as more congested patients were less likely to receive the target dose of the drugs in question at week 2 and were up-titrated more slowly [53].

The STRONG-HF study offers several clinically meaningful insights. First, despite common belief and long-lasting apprehension, the optimization of GDMT was feasible in the majority of HF patients within a relatively short period of time after HF hospitalization. This shows that the physician's attitude is a more significant and larger obstacle to achieving target doses than real, objective, physiological factors. Secondly, despite the potential harms, the rate of adverse events did not disrupt the benefits vs. risks balance (associated with adverse events). By 90 days, the incidence of any adverse event was higher in HIC (41%) when compared to the UC group (29%), but the rate of serious adverse events was similar (16% vs 17%, respectively). Apart from improving the primary composite outcome, the HIC also improved various congestion indexes and quality of life measures [54].

The STRONG-HF trial has thus shown that HIC with rapid up-titration of GDMT, even in the early post-discharge, vulnerable phase, is superior to the traditional and time-consuming step-by-step approach if performed under close follow-up and comprehensive monitoring (including NT-proBNP assessment). There are some caveats that one needs to keep in mind when interpreting the study results. STRONG-HF was an open-label study, and the HIC strategy consisted of both GDMT up-titration and frequent study visits (to ensure patients’ safety).

As it is difficult to quantify the degree of treatment optimization for patients with HF, GDMT doses achieved can be expressed as the average percentage of the target dose across medications in each class (as it was performed in STRONG-HF) or as a simple score [55]. Regardless of the method chosen, achieving higher doses of GDMT is associated with clinical benefit [56, 57].

## Decongestion related to GDMT

Without strong evidence, congestion has long been considered an important obstacle to the initiation and up-titration of GDMT, mainly treated with diuretics [58]. The diuretic-centered approach should be revisited due to several limitations, as it alleviates only symptoms and does not target the key pathophysiological processes that lead to sodium avidity and congestion development, or it can even worsen it [59, 60]. Moreover, the diuretic effectiveness is blunted in patients taking the drugs chronically, leading to the need for further escalation of diuretics, which is associated with worse outcomes [61–65]. Growing data show that the use of GDMT is related not only to improvements in survival and lower risk of HF hospitalizations but also to improvements in congestion indexes and lower loop diuretic demand [66–71]. The recently published NATRIUM-HF study has demonstrated that initiation of sacubitril/valsartan in stable ambulatory HF patients was associated with lower sodium avidity, improvements in diuretic response, and decongestion [72]. Similarly, the STRONG-HF study provides the most compelling evidence to date that intensive and comprehensive neurohormonal blockade (with RASi, beta-blocker, and MRA) initiated in the hospital, with fast up-titration of the GDMT, is associated with more effective and sustained decongestion irrespective of congestion status at randomization, with lower loop diuretic demand, and significantly lower risk of 180-day HF readmission or all-cause death. The HIC arm had significant improvements in all analyzed congestion markers, namely reductions in weight, NTproBNP, congestion score, and severity of clinical signs (JVP, pulmonary congestion, and peripheral edema) [73]. Importantly, the GDMT up-titration in STRONG-HF was started once patients were stable, without over-fluid overload, but not totally congestion-free [52]. These data, taken collectively, may challenge a "diuretic-only paradigm" of decongestion in the peri-discharge settings. High-intensive care with prompt up-titration of neuroendocrine blockade apparently translates into more effective decongestion and significantly better outcomes [74, 75].

## Patients not tolerating the target doses of GDMT

**Fig. 1:**
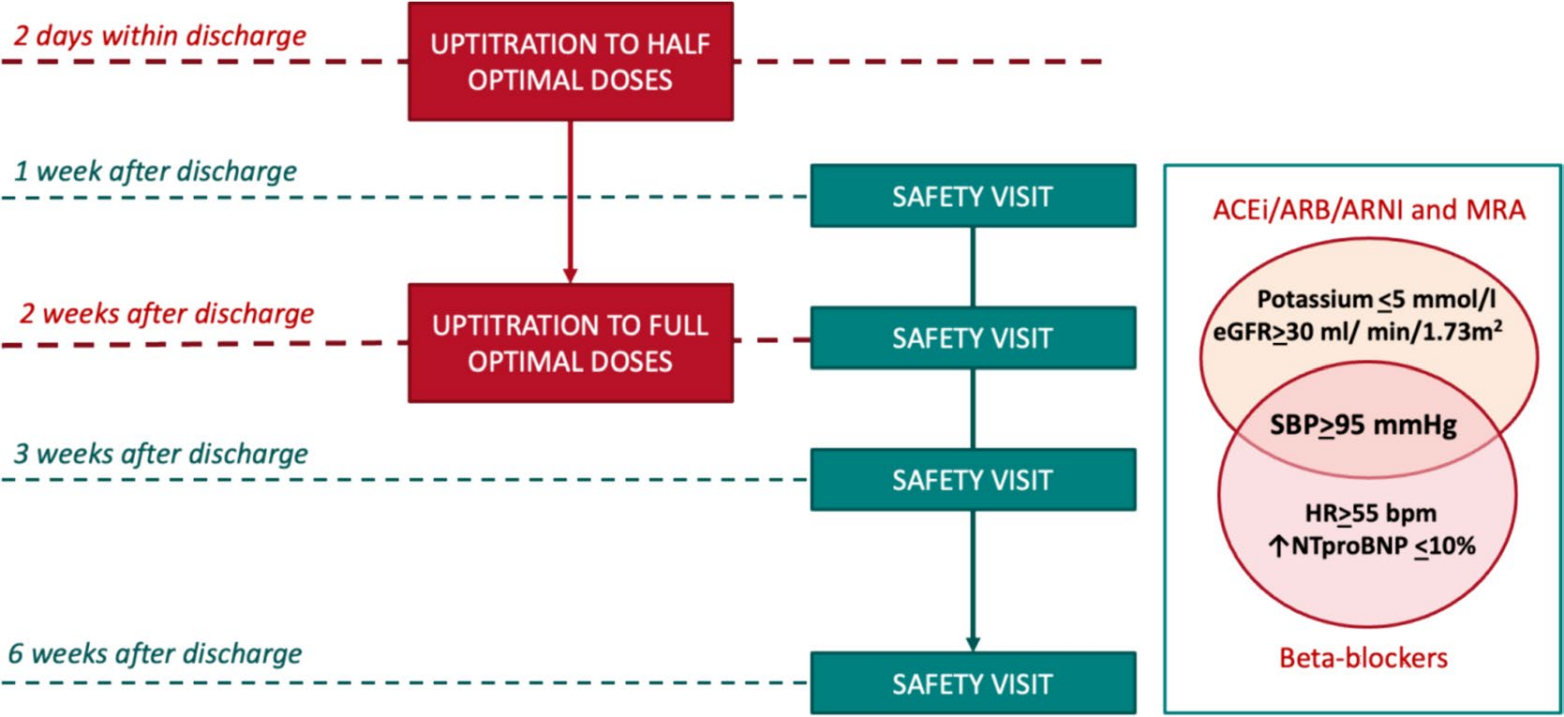
The proposed algorithm of GDMT optimization is based on a strong protocol

Beyond clinical inertia, a non-negligible proportion of patients have physiological limitations or drug intolerance, curbing full implementation of GDMT [9, 11, 76–78]. Blood pressure, heart rate, renal function, serum potassium levels, and comorbidities are common physiological barriers limiting GDMT optimization in HF. The risk of GDMT intolerance may be even higher among patients with severe HF or significant mitral regurgitation [79–81]. Identifying specific patient profiles based on these physiological factors may be helpful in tailoring GDMT and avoiding adverse events or intolerance [82]. Furthermore, it is important to identify the true “maximum tolerated dose” as well as to capture the reasons for drug intolerance after multiple attempts at drug titration [83]. Careful and strict patient monitoring and follow-up are crucial to mitigating GDMT intolerance. In STRONG-HF, rapid GDMT up-titration after hospitalization for acute HF was coupled with close follow-up to ensure patient safety during the process of medical therapy optimization [51, 52]. Multiple physiological safety indicators, including hypotension, bradycardia, worsening renal function, hyperkalemia, and increase in NT-proBNP (Fig. 1), were strictly monitored during the first six weeks after discharge, thus allowing safe and rapid GDMT up-titration (Januzzi et al., data presented at HFA 2024) [18].

Of note, decreased tolerability of neurohormonal therapy or true GDMT intolerance represents an important hallmark of the transition to an “advanced” HF stage when coupled with other factors, including recurrent HF hospitalization despite treatment optimization, worsening end-organ function, and other high-risk features [84–87]. Drugs with a minimal impact on blood pressure (i.e., SGLT2i) or novel oral inotropes without a direct effect on blood pressure (i.e., omecamtiv mecarbil or istaroxime) may be useful in patients with advanced HF, although the evaluation for potential candidacy to heart transplant or left ventricular assist device implantation remains a priority in this setting [2, 88, 89].

## The elephant in the room–ejection fraction (EF)

Ejection fraction has been an important criterion for selecting which patients should receive GDMT that relates to historical reasons. In the '60 s, '70 s, and '80 s, most HF was due to un-revascularized post-MI ischemic cardiomyopathy leading to low EF chronic HF, and hence, most HF clinics treated those patients. However, with a better treatment for ischemic heart disease, especially myocardial infarction, this type of HF has become less common, while with increasing longevity, a new type of HF has emerged – that of the elderly in which EF is commonly less reduced and sometimes normal. The latter type of HF is driven to a higher degree by neurohormonal, adrenergic, and inflammatory activation and, hence, is also in need of high-intensity care as it is associated with a high rate of adverse events [47]. For some of the medications comprising GDMT (most notably ARNi and SGT inhibitors), evidence exists that the drugs are effective and sometimes equally effective in patients with higher EF. Evidence is lacking for the others, especially BBs, although some recent studies have suggested that BBs may be effective in patients with higher EF, too. This is importantly more pronounced in acute heart failure patients, where no differences were seen in the STRONG-HF study between patients with EF above and below 40% (or 50%) with respect to the effectiveness of rapid up-titration of RASi, MRA, and BBs. Therefore, even though the evidence for high-intensity care is lacking in the chronic stage for some components of GDMT in patients with higher EF, high-intensity care should be implemented in patients with AHF before and after discharge.

## Age and comorbidities are not an excuse for not implementing high-intensity care

As shown in many studies and emphasized by STRONG-HF results, older age and comorbidities are not excuses for implementing high-intensity care. The study effect was confirmed irrespective of patients' sex, ejection fraction, and age [90–93]. Importantly, the effect of HIC on the primary outcome was present across the whole spectrum of baseline NTproBNP, but the “protective” effect seemed to be even more profound in the patients in the highest baseline NTproBNP tertile [94]. Patients who experienced an increase in NTproBNP levels after the first week of treatment had a slower GDMT up-titration but still benefited from HIC [94]. Similarly, there was no interaction between baseline eGFR and study effect. Patients who experienced a higher early decrease of eGFR had slower GDMT up-titration [95]. The number of non-cardiac comorbidities also did not limit a rapid up-titration of GDMT, nor did it attenuate the benefit of the HIC strategy on the primary endpoint. The risk–benefit ratio favored the HIC even in patients with multiple comorbidities [96]. Once the GDMT doses achieved by the patient were analyzed as the percentage of the target dose, an increase of 10% in the average percentage of the recommended dose in the HIC arm was associated with a significant reduction in HF readmission or all-cause death at 180 days (adjusted hazard ratio 0.89, 95% CI 0.81–0.98) and 180-day all-cause mortality (adjusted hazard ratio 0.84, 95% CI 0.73–0.95) [53]. High-intensity care was shown to be effective in those who were sicker either by pre-discharge MAGGIC score [90] or bio-ADM [94, 97]. Therefore, high-intensity care should be implemented in those patients as soon as possible. In those patients, as well as others, precautions should be taken to ensure that this high-intensity care is safe, but largely, there have been no major differences in the safety of high-intensity care in older patients and those with comorbidities.

## Non-adherence to high-intensive care

Non-adherence is a critical issue that needs to be addressed [23]. Finding solutions to this problem may be more significant than discovering another individual molecule that improves HF prognosis. Beyond the limitations imposed by physicians and the healthcare system, there are also obvious constraints on the patient's side, which should not be forgotten. Scandinavian databases clearly show that adherence to GDMT is a real clinical problem that translates to poorer outcomes in non-adherent patients [98, 99]. The analysis of Medicare beneficiaries' data, recently published, has shown that about half of ARNi users did not consistently adhere to the drug in the first year after starting treatment [100]. It also revealed significant racial and socioeconomic disparities in long-term adherence to ARNi [100]. Furthermore, fatigue from the illness and its management, depression, and financial burden can be reasons for non-adherence to the prescribed treatment. The concept of the polypill may address some of the drawbacks associated with polypharmacy burden. There are other potential future solutions that should increase adherence, including finding depot formulas for GDMT that release active molecules over a substantial period of time or using drugs that can be given as antibodies once a month. These drugs could include anti-ACE or anti-adrenergic antibodies that are currently being developed. Some side effects, such as hypotension, eGFR drop, or hyperkalemia, may also disrupt adherence to GDMT. Instead of withdrawing MRA or RASi, using potassium binders may be a solution for the latter. However, it needs to be prospectively tested whether this approach leads not only to a decrease in serum potassium but also to improvement in outcomes [76, 101]. Lastly, the coadministration of some safe inotropic/vasoactive agents to patients with low blood pressure to increase their tolerability of GDMT is also a potential solution to increase pharmacological adherence in HF.

## Remote monitoring

Remote monitoring of HF patients, encompassing both invasive and non-invasive methods, is a rapidly advancing field aimed at improving patient outcomes and reducing hospitalizations [102]. This approach also aims to increase patients’ quality of life and reduce the disease-related burden (outpatient visits, etc.). Invasive monitoring typically involves implantable devices such as CardioMEMS, which measure pulmonary artery pressure, providing early detection of worsening HF. These devices offer accurate, continuous data but require invasive procedures for implantation. The data available confirms that treatment based on information derived from those devices can lead to a decrease in the risk of heart failure (HF) events [103–105]. However, it is crucial to understand that telemonitoring can help physicians identify the clinical problem, but the outcome is directly related to the actions taken by the physician or patient [106]. On the other hand, non-invasive monitoring employs wearable technologies, mobile apps, and home-based sensors to track vital signs like heart rate, blood pressure, and weight. Non-invasive methods are more convenient and accessible, reducing the need for hospital visits and allowing for timely interventions through telemedicine. Together, these approaches facilitate proactive HF management, improving quality of life and potentially decreasing healthcare costs through early detection and intervention [107].

## The future of high-intensity care in HF

At the moment, the recommended doses of GDMT are derived from pharmacologic studies, which have established effective and safe medication dosages. However, studies implementing rapid simultaneous up-titration of the four pillars of GDMT have shown that this approach is feasible and leads to reverse remodeling and better outcomes [52, 53]. There should be no hesitation or perceived barriers to such implementation as soon as suboptimal therapy has been identified, either in the outpatient setting or during an AHF admission. In the near future, it may be possible to identify certain clinical/laboratory markers of treatment efficacy/goal attainment (individually) through which therapy could be individualized. Furthermore, it can be envisioned that specific constellations of markers in response to dosing might also provide a prognostic signal. Moreover, the drugs that target novel pathophysiological pathways, like anti-inflammatory therapies, anti-fibrotic drugs, novel inotropes, or drugs that improve energetic pathways, may provide further benefit in HF.

## How to implement high-intensity care?

Regretfully, high-intensity care implementation has been left to the discretion of the treating physicians, many of whom are not only not HF experts but, in many cases, are not even cardiologists. In the setting of a general practitioner clinic, the treating physician is commonly busy and does not have the band with or means to implement high-intensity care for HF patients. A recent study has shown that this high intensity can be easily implemented by non-physicians, leading to significantly improved patient status. Therefore, we call on the scientific community, healthcare providers, insurance companies, and governments to urgently revamp the care of HF patients. These patients, who have adverse outcomes matching and sometimes surpassing those of oncological patients, should be cared for in specialized centers by specialized teams, the same as cancer patients. This care should include rapid up-titration of GDMT, addressing financial, socioeconomic, and non-adherence barriers to care, and implementing intensive remote monitoring. These centers should be comprised of a multidisciplinary team including nurses, doctors, pharmacists, and even mental health providers, as mental health is commonly comorbid with HF (Januzzi et al., data presented at HFA 2024). Such care, as recently shown, can be implemented successfully and improve substantially the health status of HF patients.
